# A Barcoding Strategy for Pooled Single-Cell LA-ICP-TOFMS Analysis of Metal-Containing Therapeutics

**DOI:** 10.1021/acs.analchem.6c01916

**Published:** 2026-06-29

**Authors:** Claude Molitor, Lyndsey Hendriks, Antonia Hafner, Bernhard Keppler, Walter Berger, Gunda Koellensperger

**Affiliations:** † Institute of Analytical Chemistry, Faculty of Chemistry, 27258 University of Vienna, Vienna 1090, Austria; ‡ Institute of Inorganic Chemistry, Faculty of Chemistry, University of Vienna, Vienna 1090, Austria; § Vienna Doctoral School in Chemistry (DoSChem), University of Vienna, Vienna 1090, Austria; ∥ Center for Cancer Research, 89097 Medical University of Vienna, Vienna 1090, Austria; ⊥ Comprehensive Cancer Center, Medical University and General Hospital of Vienna, Vienna 1090, Austria

## Abstract

Laser ablation inductively coupled plasma time-of-flight mass spectrometry (LA-ICP-TOFMS) enables the quantitative imaging of metal-based therapeutics with cellular resolution. Despite its analytical power, systematic screening of metal-containing anticancer agents remains limited by the substantial time, cost, and labor required for cell handling, staining, and analysis. Here, we present a barcoding strategy that allows us to pool multiple experiments together and analyze them simultaneously. Our labeling approach combines wheat germ agglutinin (WGA) with lanthanide-labeled anti-WGA barcodes, enabling robust discrimination of experimental conditions. Following LA-ICP-TOFMS measurement, pooled data sets can be processed using MeXpose, a data processing pipeline for single cells, to accurately assign each cell to its original barcode, i.e., experiment. In this study, we applied this novel strategy to investigate the uptake of BOLD-100 and oxaliplatin in HCT116 wild-type (WT) and oxaliplatin-resistant (OxR) colorectal cancer cells using different drug concentrations. Pooling 10 experiments into a single analytical run allowed us to reduce consumable use (mainly argon for the ICP and antibody usage if stained), measurement time, and downstream processing while increasing data consistency. Barcoding-enabled single-cell analysis confirmed a substantial reduction in oxaliplatin uptake in oxaliplatin-resistant HCT116 cells compared with the WT cell line, whereas BOLD-100 uptake was affected to a much lesser extent. These results demonstrate the utility of this strategy for the efficient and scalable assessment of metal-based therapeutics.

## Graphical abstract

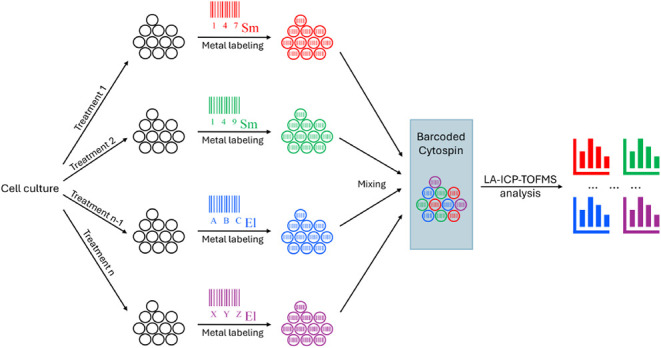

## 1 Introduction

Barcoding in analytical chemistry refers to a multiplexing strategy that enables the pooled preparation, measurement, and processing of multiple samples within a single analytical run. This approach addresses the often rate-limiting issue of analytical throughput, particularly in omics applications, and has gained significant momentum with the advent of multiplexed single-cell analyses.^1–3^

Today, scalable single-cell technologies drive some of the most exciting discoveries in biology and medicine.^4–6^ Cytometry, an especially impactful platform, enables the rapid characterization of cellular phenotypes, with suspension-based systems capable of analyzing thousands of cells per second. Cell suspensions containing 10^5^–10^6^ intact cells can therefore be rapidly interrogated at the single-cell level. However, sample-to-sample switching remains challenging, making barcoding essential for increasing the analytical throughput. Early flow cytometry (FC) studies combined three fluorescent cell barcodes at different concentrations to generate unique signatures for up to 96 samples.^7^ With the introduction of mass cytometry (MC) nearly two decades ago, the number of measurable parameters per cell increased significantly.^2^ This technique is based on inductively coupled plasma time-of-flight mass spectrometry (ICP-TOF-MS) and uses antibodies conjugated to polymer-based metal tags instead of fluorophores, substantially increasing the number of potential readouts per cell.^8^ As a consequence, error-prone FC barcoding strategies relying on quantitative label-intensity patterns could be substituted with more selective and robust approaches,^9–12^ using either single-barcode schemes or customized multiplexed barcodes.^11–13^ The barcode designs either allow for broad application and do not feature any cell-type specificity^10–13^ or are cell-type specific. A widely applied strategy allows barcoding of 35 whole blood samples by permeabilization-free labeling with the CD45 antibody.^9^

The power of barcoding fully leverages mass cytometry; however, it has also been successfully applied to imaging mass cytometry (IMC).^14^ In a seminal IMC study, multiplex barcoding^11^ was used to quantify the phenotypic and signaling states^15^ of cells in multiple spheroids at high throughput.^16^ By pooling multiple spheroid samples into one single imaging measurement, acquisition time was reduced, and limitations related to sample holder capacity were mitigated. Additionally, the IMC sample preparation protocol could be applied to the pooled spheroids, significantly reducing the volume of the required antibody stains.

Single-cell analysis based on ICP-MS not only enables phenotypic profiling but also allows for the quantification of metal content at the single-cell level. Multielemental patterns can be used to classify distinct cell types in heterogeneous populations^17,18^ or to characterize metal heterogeneity within a single-cell population.^18,19^ Fully leveraging the potential of MC/IMC, quantitative metal accumulation can be linked to cell type, function, and state. This concept has been successfully applied to metal exposure studies involving toxic metals and metal-based drugs, both of which form macromolecular complexes within tissues and cells.^20,21^

In this work, we introduce a barcoding strategy tailored for high-throughput screening of metal accumulation in single cells using laser ablation ICP-TOFMS. As a proof of principle, the accumulation of two metal-based anticancer drugs, namely oxaliplatin and sodium *trans*-[tetrachloridobis­(indazole)­ruthenate­(III)] (BOLD-100), was investigated across multiple experimental conditions. Assessing intracellular drug accumulation *in vitro* is a critical step in the development of metal-based anticancer therapeutics. Single-cell measurements enable the detection of heterogeneity that is often obscured in bulk analyses, for example, when small subpopulations with distinct phenotypes emerge upon treatment.

The proposed screening platform is based on laser ablation ICP-TOF-MS, where quantification of metal-based drugs can be achieved through external standardization using gelatin microdroplets. Using this workflow, hundreds of single cells can be imaged in minutes. The validity of this calibration strategy has been demonstrated previously, and cross-validation with quantitative solution-based single-cell measurements has confirmed the suitability of this bioimaging approach for measuring metal-based drug accumulation in single cells.^22,23^

The envisioned application of quantitative bioimaging imposes specific requirements on barcode design, including the avoidance of cell permeabilization and the need to support robust cell segmentation. As LA-ICP-TOF-MS continues to advance toward higher spatial resolutions (250–600 nm),^24–26^ smaller ablation spot sizes result in reduced material removal and lower detected signal intensities. In addition, antigen retrieval steps associated with antibody staining can further attenuate membrane signals. Under these conditions, intense and well-defined membrane labeling becomes essential for accurate segmentation and reliable barcode detection.

The dual-label WGA/anti-WGA strategy presented here was originally developed as a membrane marker^24^ and provides a high-intensity, surface-localized signal that enables clear delineation of cell boundaries. Fluorescently labeled WGA binds specifically to N-acetylglucosamine residues on the cell surface and is subsequently recognized by metal-conjugated anti-WGA antibodies generated using standard lanthanide labeling protocols. By conjugating anti-WGA antibodies with different metal isotopes, this approach can be repurposed for barcoding, allowing a single reagent to support both cell segmentation and sample assignment.

The introduced 1-plex WGA/anti-WGA strategy enables accurate segmentation and reliable barcode readout under state-of-the-art imaging conditions (1 μm), while allowing straightforward exclusion of mis-segmented cells or doublets. Unlike conventional barcoding approaches, this strategy eliminates the need for cell permeabilization and is not restricted to a limited set of isotopes, such as Pd or Te. Instead, metal isotopes can be freely selected, enabling flexible panel design. Moreover, the approach readily extends to multiplexed barcoding, as combinations of metal-labeled anti-WGA conjugates can be used to generate custom X-choose-Y barcode sets tailored to the isotopic channels available within a given antibody panel.

In addition to barcode design, several practical factors must be considered when applying barcoding strategies to single-cell LA-ICP-TOFMS imaging. Barcode signal assignment may be affected by spectral interferences, as well as lateral signal spread arising from finite pixel resolution or occasional line-shift artifacts, which occur during image acquisition. These aspects are, therefore, examined as part of the validation of the barcoding workflow presented in this study.

## 2 Experimental Section

The overall workflow for barcoding, pooling, and sample preparation of samples for LA-ICP-TOFMS analysis is illustrated in Figure 1.

**Figure 1:**
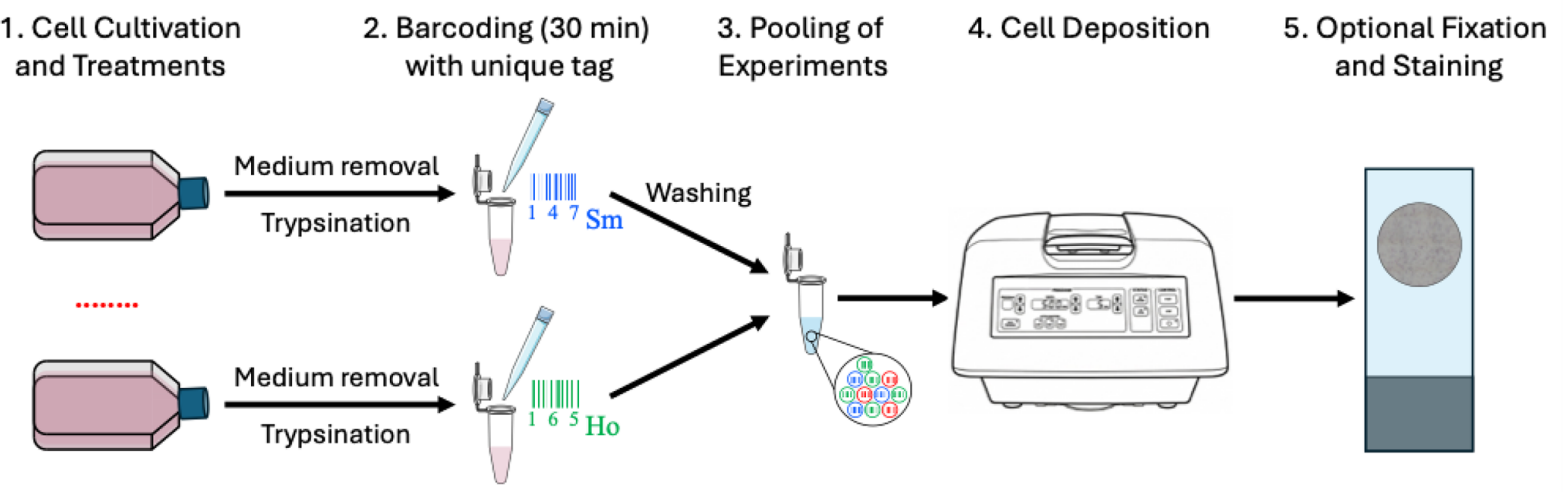
Overview of the barcoding-based sample preparation procedure for pooled LA-ICP-TOFMS. (1) Cells are cultured and treated under different experimental conditions. (2) Each condition is labeled with a unique barcode. (3) Barcoded samples are pooled into a single experiment. (4) Cells are deposited on a glass slide by cytospin technology. (5) The cells are fixed and stained for analysis, if needed.

### 2.1 Chemicals and Materials

Ultrapure water was produced using a Purelab Ultra MK 2 system (ELGA, High Wycombe, U.K.). Bovine serum albumin (BSA) and Tris-buffered saline (BioUltra) were purchased from Sigma-Aldrich (Steinheim, Germany). Human BD Fc Block was supplied by BD Biosciences (San Jose, CA, USA). The Tris/EDTA-based Dako Target Retrieval Solution (pH 9) was obtained from Agilent Technologies (Waldbronn, Germany). Tween 20 and SuperBlock blocking buffer (TBS) were purchased from Thermo Fisher Scientific (Waltham, MA, USA).

The Cell-ID Ir-intercalator (125 μM) was acquired from Standard BioTools (San Francisco, CA, USA). Anti-Wheat Germ Agglutinin (anti-WGA; Szabo Scandic, Vienna, Austria) was metal-tagged using Maxpar X8 Antibody Labeling Kits (Standard BioTools) with the following lanthanides: ^143^Nd, ^144^Nd, ^145^Nd, ^147^Sm, ^149^Sm, ^151^Eu, ^152^Sm, ^154^Sm, ^155^Gd, and ^165^Ho. Final concentrations of the metal-conjugated anti-WGA antibodies (M-anti-WGA) were approximately 2 mg/mL. DAPI staining solution (1 μg/mL) and FITC- and Rhodamine-conjugated WGA (5 mg/mL) were obtained from GeneTex (Irvine, CA, USA).

### 2.2 HCT116 Cell Cultivation and Drug Treatment

The human colorectal cancer cell line HCT116 wild type (WT) was obtained from the American Type Culture Collection (ATCC). An oxaliplatin-resistant derivative (HCT116 OxR) was generated in-house by stepwise exposure of HCT116 WT cells to increasing concentrations of oxaliplatin, with resistance maintained by applying a 10 μM oxaliplatin pulse every 2 weeks. Cells were cultured in McCoy’s 5A medium supplemented with 10% v/v heat-inactivated fetal calf serum (FCS; BioWest) and 2 mM l-glutamine (VWR International) at 37 °C in a humidified atmosphere containing 5% CO_2_.

For experiments, cells were seeded either in T25 flasks (Greiner Bio-One) at a density of 2 × 10^6^ cells in 5 mL medium or in T75 flasks at 5 × 10^6^ cells in 10 mL medium. After the cells were allowed to adhere overnight, treatments were applied for 16 h according to the conditions listed in Table S1. BOLD-100 was provided by the University of Vienna (Institute of Inorganic Chemistry, University of Vienna),^27^ and oxaliplatin was purchased from LC Laboratories (Oxaliplatin; OxPt; LC Laboratories; #O-7111).

Following treatment with BOLD-100 or Oxaliplatin, cells were detached using Trypsin/EDTA (Sigma-Aldrich), centrifuged at 1400 rpm for 5 min, the supernatant was discarded, and pellets were resuspended in 120 μL TBS.

### 2.3 HeLa Kyoto Cell Cultivation and Drug Treatment

HeLa Kyoto cells were grown in Dulbecco’s medium and then split into two aliquots. One aliquot served as a control, while the other was treated with the taxane chemotherapy agent docetaxel (DTX) for 3 days.^28^ Each aliquot was then subdivided. One fraction was fixed by using 4% v/v paraformaldehyde for 10 min and resuspended in TBS. The second contained living cells and was washed with TBS prior to barcoding.

### 2.4 Optimized Barcoding Procedure

Approximately 1.5 × 10^6^ cells per condition were resuspended in 120 uL TBS. Fluorescent WGA (FITC- or Rhodamine-WGA) and metal-labeled anti-WGA (M-anti-WGA) were preincubated for 2 h at equimolar concentrations in a volume of 30 μL. For the F-WGA and M-Anti-WGA used here, this corresponded to a dilution of 1:25 for FITC-WGA and 1:25 for Anti-WGA during preincubation. After 2 h, the 30 μL barcoding mix was then added to the 120 μL cell suspension and incubated for 30 min with occasional vortexing. This corresponds to a final M-Anti-WGA dilution of 1:50 during cell staining. Cells were pelleted (1400 rpm, 5 min), washed twice with TBS, resuspended in 1.5 mL TBS, and pooled across conditions where indicated. Cells were deposited onto Superfrost microscope slides (Thermo Scientific) using a cytospin cytocentrifuge (Shandon Cytospin 4, Thermo Scientific, UK) at 400 rpm for 5 min. Cytospins were air-dried for at least 20 min and subsequently fixed with 4% v/v PFA using 80 μL per spot for 20 min at room temperature (RT). Slides were rinsed with ultrapure water and stored at −20 °C until further analysis.

### 2.5 Immunostaining of Cytospins

Heat-induced antigen retrieval was carried out in Tris–EDTA buffer (pH 9) at 96 °C for 30 min. After cooling to RT, samples were washed with ultrapure water, followed by TBS containing 0.05% v/v Tween 20 to achieve permeabilization. To minimize nonspecific antibody binding, sections were incubated with SuperBlock for 30 min at RT and subsequently treated with Human BD Fc Block for 10 min.

Primary antibody staining was performed overnight at 4 °C in a hydration chamber, followed by two washes with TBS/0.05% v/v Tween 20 under gentle agitation. DNA staining was performed in a hydration chamber by incubating the sample for 5–10 min with DAPI in combination with the iridium-based DNA intercalator (125 μM). After two additional washes with TBS/0.05% v/v Tween 20, samples were rinsed with ultrapure water. Stained cytospins were subsequently examined by immunofluorescence microscopy and analyzed using LA-ICP-TOFMS.

### 2.6 DNA Staining of Cytospins

For cytospins subjected to DNA-staining only, the samples were incubated for 5 min with a DAPI staining solution in combination with the iridium-based DNA intercalator (125 μM). Similarly, samples were rinsed with ultrapure water and subsequently examined by immunofluorescence microscopy and analyzed using LA-ICP-TOFMS.

### 2.7 Immunofluorescence Microscopy Analysis

Stained cytospins were subsequently examined by immunofluorescence microscopy using an Axioscope 7 microscope equipped with a Colibri 7 light source (Zeiss, Oberkochen, Germany) and a 20× objective. Fluorescence signals were detected using the following excitation filters (center wavelength/bandwidth): 380/30 nm for DAPI, 469/38 nm for FITC, and 511/44 nm for Rhodamine.

### 2.8 LA-ICP-TOFMS Analysis

LA-ICP-TOFMS measurements were carried out using an Iridia 193 nm laser ablation system (Teledyne Photon Machines, Bozeman, MT, USA) with the Aerosol Rapid Introduction System (ARIS) to ensure efficient and reproducible aerosol transport, coupled to an ICP-TOFMS (icpTOF 2R, TOFWERK, Thun, Switzerland). Instrument performance was optimized daily using NIST SRM 612, adjusting for high sensitivity, low oxide formation (<2.5% for ^232^Th^16^O^+^/^232^Th^+^), and minimal elemental fractionation (^238^U/^232^Th = 1–1.5%). A detailed overview of all operating parameters is provided in Table S2. Ablation was performed with circular spot sizes of 2–3 μm, using an interspot spacing of 1 μm. A fixed dosage of 2–3 was applied, resulting in 2× to 3× overlap in both *x* and *y* directions to ensure complete material removal. Consequently, all images were acquired with an effective pixel size of 1 μm.

### 2.9 Data Acquisition and Processing

LA-ICP-TOFMS measurements were recorded using TofPilot software (version 2.10.3.0; TOFWERK AG) with the laser ablation module, and raw data were saved in HDF5 format. Data reconstruction and handling were performed in MeXpose.^29,30^ Segmentation was performed using Cellpose3 or Cellpose-SAM.^31,32^ Image processing was done in Fiji (v2.16.0/1.54p, NIH).^33^

## 3 Results and Discussion

### 3.1 Specificity of the Dual Label for Fixed and Living Cells and Optimization of the Barcoding

The WGA/anti-WGA membrane labeling method supports fluorescence imaging through either rhodamine- or FITC-labeled WGA, together with mass spectrometric imaging through the metal-labeled secondary anti-WGA antibody. This dual-labeling capability enables the evaluation and optimization of a barcoding protocol with high stability throughout sample pooling and resuspension, suitable for both fixed and living cells following drug exposure.

Four experimental conditions of HeLa cells were labeled without cell permeabilization, pooled into one sample for cyto-spinning, and imaged by fluorescence microscopy and LA-ICP-TOFMS (2 μm circular spots, dosage 2, and interspacing of 1 μm, at 500 Hz). Each experimental condition was encoded using a dual-labeled barcode consisting of fluorescently labeled WGA and metal-labeled anti-WGA antibody as follows: (1) untreated living cells: FITC-WGA/^149^Sm-anti-WGA; (2) untreated paraformaldehyde (PFA)-fixed cells: FITC-WGA/^151^Eu-anti-WGA; (3) treated living cells: rhodamine-WGA/^152^Sm-anti-WGA; and (4) treated PFA-fixed cells: rhodamine-WGA/^165^Ho-anti-WGA. As can be observed in Figure 2, a clear separation between untreated and treated cells was obtained based on stacked isotope signals (^149^Sm and ^151^Eu in green for untreated cells, and ^152^Sm and ^165^Ho in blue for treated cells). Moreover, the high spatial correlation between the two imaging modalities indicated a high specificity and stability of the WGA/anti-WGA complex under both live-cell and fixed-cell conditions. The presented approach offers simultaneous barcoding and membrane labeling for segmentation. Segmentation based on fluorescence and metal barcodes yielded nearly identical results, with comparable cell counts (630 and 617 cells, respectively) and similar average cell sizes (332 μm^2^ and 349 μm^2^). To enable a direct comparison, the fluorescence image was manually cropped to match the field of view of the LA image. Cells touching the image border were excluded from image analysis. Taken together, the high spatial correlation of fluorescence and mass spectrometry signals, along with the good agreement in cell count and size, confirms the validity and robustness of the proposed barcoding strategy.

**Figure 2:**
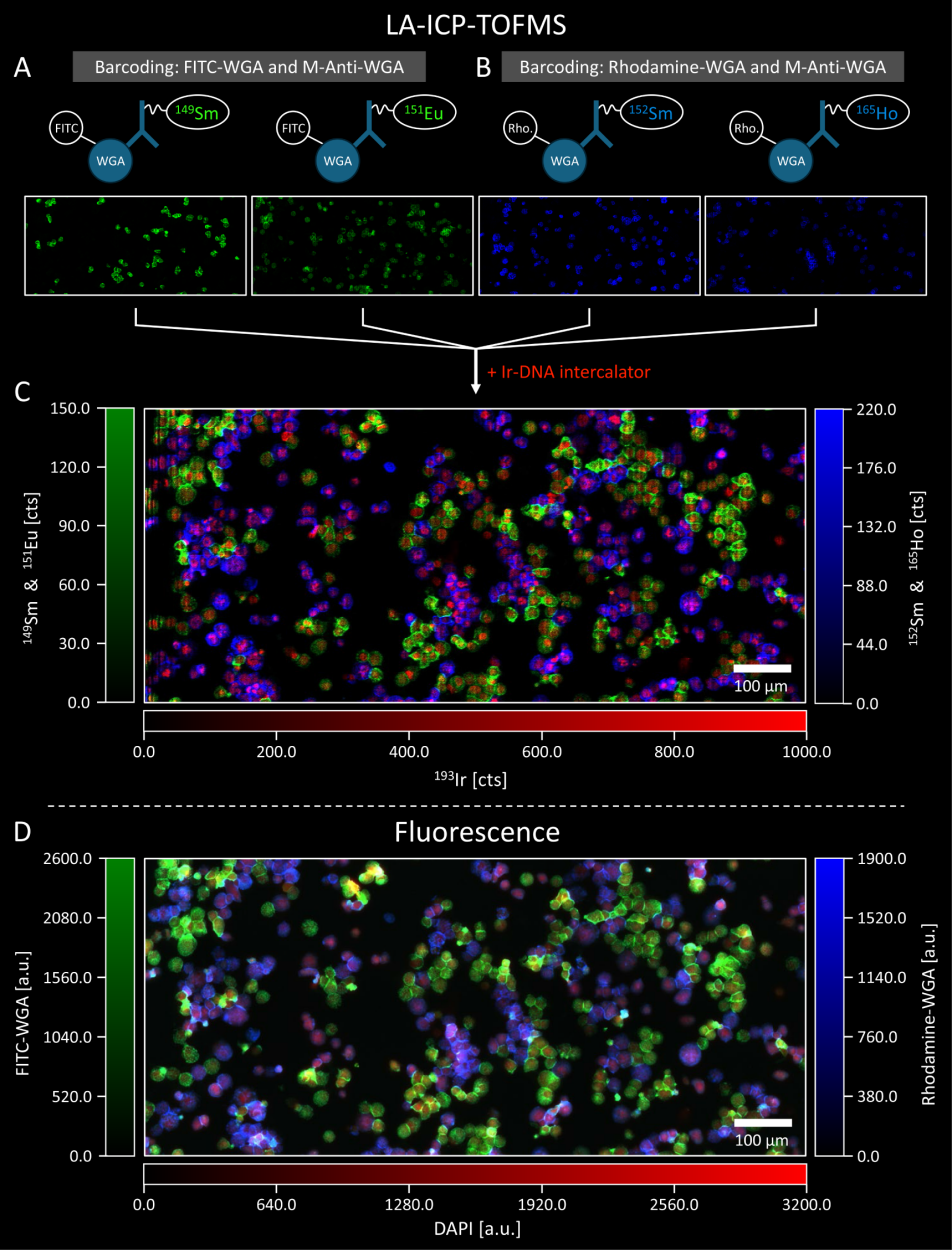
Dual-label WGA/anti-WGA membrane barcoding visualized by fluorescence microscopy and LA-ICP-TOFMS. (A) Control samples barcoded with FITC-WGA: living cells with ^149^Sm-anti-WGA and PFA-fixed cells with ^151^Eu-anti-WGA. (B) Treated samples barcoded with Rhodamine-WGA: living cells with ^152^Sm-anti-WGA and PFA-fixed cells with ^165^Ho-anti-WGA. (C) For visualizing the high spatial correlation between the two imaging modalities, the ^149^Sm and ^151^Eu channels ((A)­FITC-WGA-labeled) were stacked into one green channel; the ^152^Sm and ^165^Ho channels ((B) Rhodamine-WGA-labeled) into one blue channel. The third channel, in red, comprises an ^193^Ir-DNA intercalator. (D) Corresponding fluorescence images show FITC (green), Rhodamine (blue), and DAPI as the DNA marker (red). Elemental maps were acquired with LA-ICP-TOFMS at 1 μm pixel resolution (2 μm circular spot size, 2× overlap in both directions) with a repetition rate of 500 Hz.

To further optimize the F-WGA/M-anti-WGA barcoding protocol, a dilution series of the dual-label was applied to cancer cells obtained as aliquots from a single *in vitro* experiment (see Figure S1). HCT116 colon cancer cells exposed to 5 μM oxaliplatin for 16 h served as a model system. Two aliquots were used for quality control: a nonbarcoded sample and a procedural blank prepared following the barcoding steps but using buffers only (Figure S1A and B). The remaining three aliquots comprised a three-point concentration series, with final M-anti-WGA dilutions of 1:150, 1:50, and 1:30 during the 30 min barcoding incubation.

At high dilution (1:150), the membrane labeling was poor (see Figure S1C). A dilution of 1:50, corresponding to an M-Anti-WGA concentration of 40 μg/mL, produced clearly defined membrane borders at cell apposition sites, improving boundary delineation and segmentation accuracy (see Figure S1D). Further increasing the antibody concentrations did not yield additional improvements (see Figure S1E). Using 3 μL of M-Anti-WGA for the 1:50 dilution was sufficient to stain 1.5 × 10^6^ cells (∼15 cytospins).

Finally, to assess potential effects of the barcoding procedure on drug retention, single-cell platinum signals were evaluated in the HCT116 WT sample set. All five samples were stained for 5 min using an Ir-DNA intercalator. Segmentation was performed on the stacked barcode isotope and ^193^Ir signal for barcoded samples. For the nonbarcoded sample and procedural control, the ^63^Cu and ^193^Ir signals were used. The differences in single-cell Pt accumulation were addressed in HCT116 cells by comparing the nonbarcoded samples and the samples barcoded with different concentrations of anti-WGA. Across the dilution series, no significant Pt loss was observed, indicating that neither the barcoding procedure (with a duration of 45 min) nor moderate increases in WGA concentration induced a measurable bias (Figure S2A). In contrast, a distinct decrease of Pt quantities was observed in HCT116 WT cells exposed to 10 μM oxaliplatin (16 h), which were labeled according to the protocol but left suspended in TBS for 2 h before cytospin preparation (Figure S2B), emphasizing the need for controlled, rapid sample preparation protocols.

Barcoding substantially mitigates the problem by pooling all of the experimental conditions into a single tube. All cells undergo identical handling steps and exposure times, which not only minimize the resting time in solution but also eliminate intersample timing variability. This results in increased data robustness and comparability across conditions. Finally, the segmentation performance was notably improved in the barcoded samples. Nonbarcoded cytospins lack a dedicated membrane marker and therefore require lower cell densities to avoid touching cells. This reduces the number of analyzable cells per unit area. The WGA/anti-WGA barcode enables denser cytospins, approximately three- to four-fold higher cell counts per area, without compromising segmentation accuracy.

Together, these experiments establish 1:50 as an optimal compromise among signal intensity, reagent consumption, and segmentation performance for the F-WGA/M-anti-WGA barcoding strategy.

### 3.2 Barcoding and Debarcoding of a Pooled Data Set

#### 3.2.1 Experimental Data Set and Barcoding Scheme

After optimization of the WGA/anti-WGA barcoding workflow, the method was applied to a pooled *in vitro* data set to evaluate its performance for multicondition single-cell drug uptake studies. Next to the clinically established drug oxaliplatin, a candidate ruthenium drug (BOLD-100, sodium *trans*-[tetrachloridobis­(indazole)­ruthenate­(III)]) was tested. Experiments were conducted in the colon cancer model HCT116, including the oxaliplatin-sensitive wild-type cell line (HCT116 WT) and the oxaliplatin-resistant subline (HCT116 OxR). In total, 10 *in vitro* experiments were generated, including control samples (for experimental details, see Figure 3 and Table S1). Each experimental condition was encoded using a 1-plex barcode, consisting of one of the following 10 isotopes: ^143^Nd, ^144^Nd, ^145^Nd, ^147^Sm, ^149^Sm, ^151^Eu, ^152^Sm, ^154^Sm, ^155^Gd, and ^165^Ho.

**Figure 3:**
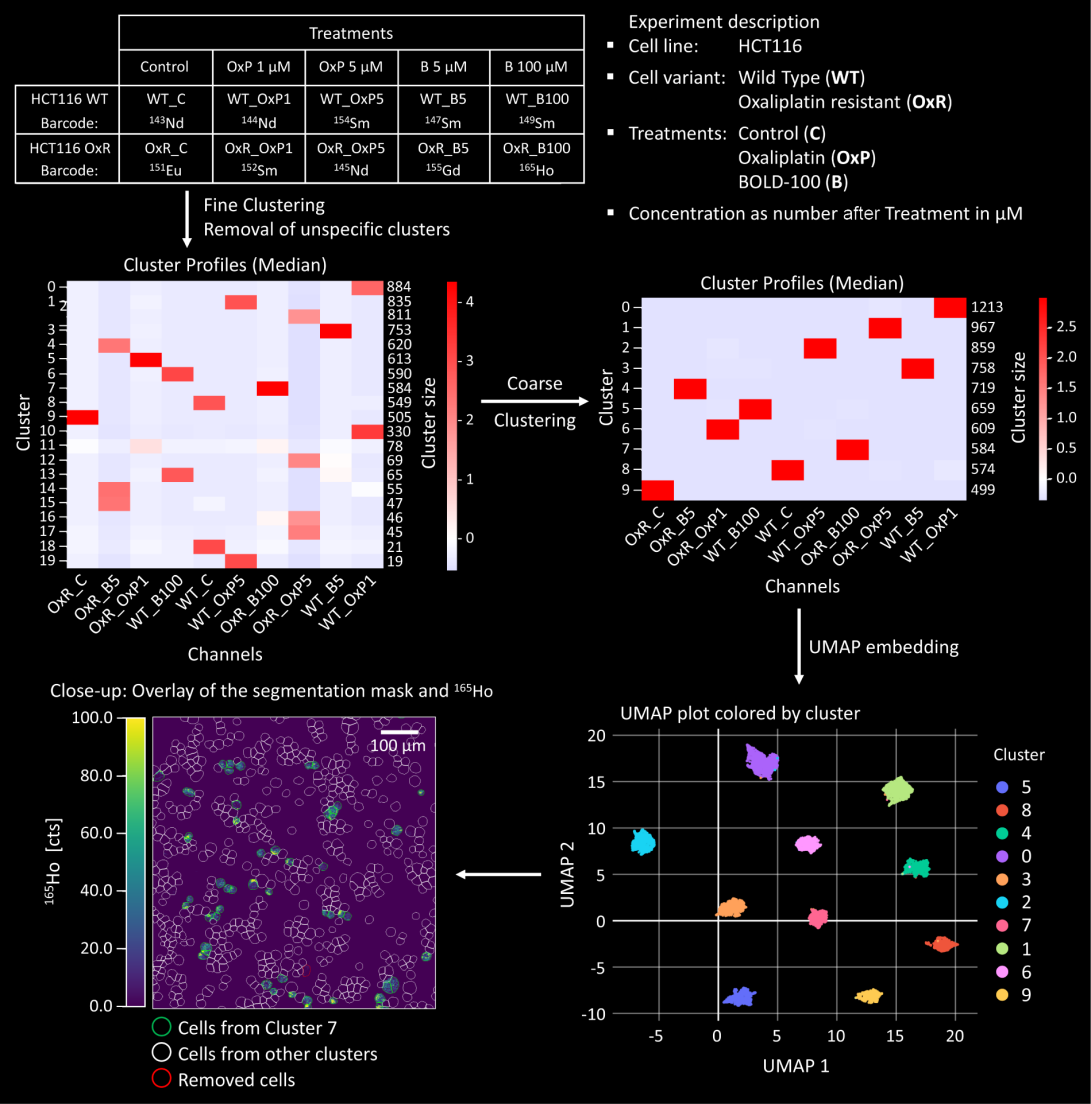
Debarcoding workflow based on unsupervised Leiden clustering for 10 experimental conditions includes: (1) fine clustering to remove nonspecific clusters or doubly positive clusters. (2) Coarse clustering and embedding of remaining data points based on barcode expression. (3) Cropping of the measured signal to display the overlay of cells attributed to Cluster 7 (green masks) with the respective barcode in viridis (^165^Ho), cells from different clusters (white masks), and cells removed during fine clustering (red masks). Maps were acquired with LA-ICP-TOFMS at 1 μm resolution (2 μm circular spot size, 2× overlap in both directions) with a repetition rate of 500 Hz.

#### 3.2.2 Segmentation and Debarcoding

For segmentation (Cellpose-SAM),^31^ all barcodes were stacked into one single layer to obtain a uniform membrane label, with the ^193^Ir-DNA signal as a secondary channel. The segmented cell data were submitted to different debarcoding methods.

In the first step, debarcoding was accomplished by density-plot-driven manual gating. Gating was applied to the barcode channels across the entire initial cell population for every gate rather than sequentially on residual populations. This approach intentionally exposes ambiguous assignments: among 7520 analyzed cells, 212 cells (2.8%) could not be assigned to any gate, and 128 cells (1.7%) were assigned to two or more gates, indicating barcode patterns inconsistent with any valid key. Such ambiguous events were flagged and excluded from downstream analysis. Figure S3 shows a UMAP embedding of cells based on arcsinh-normalized barcode signals, overlaid with density-plot-derived gates as color annotations. The UMAP embedding demonstrates clear separation of experimental conditions, consistent with the manual gates.

To overcome the challenges of manual gating, we implemented an alternative debarcoding strategy based on unsupervised Leiden clustering by PhenoGraph, as provided in MeXpose.^29,30^ In contrast to density-plot gating, a pairwise (duplex) approach that compares two channels at a time, unsupervised Leiden clustering considers the full multivariate barcode feature space. This enables a faster, fully multiplex decision that is independent of manual thresholds and, thus, is more reproducible. Minor barcode signal bleed into neighboring cells is largely negligible after transformation and scaling, since high-intensity barcode channels dominate the distance metrics and preserve correct cluster assignment. While unsupervised Leiden clustering by PhenoGraph is best known in IMC to group cells into populations based solely on multivariate marker expression, the same principle can be applied to barcode channels. Cells that share a characteristic marker expression pattern are grouped into coherent populations (e.g., blood vessels, fibroblasts, basal and muscle cells in skin);^30^ the same logic applies to debarcoding, where cells from the same experimental condition cluster because they share a common signal expression based on the barcode intensity. In both cases, cells are grouped by similarity in their marker expression, whether phenotypic markers or barcode isotopes.

Within the MeXpose2 pipeline,^29,30^ barcode-based clusters are computed on arcsinh-normalized barcode signals and can be exported for downstream analysis. Cluster granularity can be adjusted first by lowering the k-parameter to obtain finer communities and isolate ambiguous or weakly labeled cells. In this data set (k = 15), 19 clusters were identified. Cluster 11 (*n* = 78, ≈1% of all cells) showed no enrichment of any barcode and was therefore marked as unassigned and removed. The remaining cells were either grouped according to their shared barcode profiles in the heatmap or subjected to reclustering (k = 200 for this data set) and subsequent embedding. The accompanying heatmap and UMAP in Figure 3 illustrate the clear separation of barcode-defined populations. Spatial validation confirmed correct assignment, as cells attributed to a given cluster colocalized with the corresponding barcode channel in the reconstructed images (Figure 3 overlay).

#### 3.2.3 Applied Case Study: Drug Uptake Analysis

Applying the WGA/anti-WGA pipeline to an *in vitro* study with two metal-based drugs and different conditions (see Figure 3) revealed that barcoding-specific spillover was negligible. Ten experiments were debarcoded using unsupervised Leiden clustering by PhenoGraph, yielding 10 clusters ranging from 499 to 1213 cells. The barcode isotope ^149^Sm showed a mean of 13,924 ± 5990 cts/cell in the WT_B100 condition, compared to 186 ± 597 cts in the respective control (WT_C), confirming that barcoding-related spillover remained negligible relative to true barcode intensities. WT_OxP5 exhibited the most significant accumulation of ^196^Pt with a mean of 81.1 ± 45.4 cts per cell compared to 5.0 ± 3.1 cts per cell in the corresponding control (WT_C). The highest ^102^Ru accumulation was observed in WT_B100, reaching 445.3 ± 226.4 cts per cell compared to 13.4 ± 19.0 cts per cell in the respective control (WT_C).

Following debarcoding and data cleanup with MeXpose^29,30^ (Figure 3), metal-based drug accumulation was compared between WT and OxR HCT116 cells to assess how oxaliplatin resistance influences platinum and ruthenium uptake. Violin plots of one barcode, ^102^Ru and ^196^Pt content per cell across all conditions are within Figure S4. Briefly, oxaliplatin uptake was notably reduced in HCT116 OxR compared to the WT at 1 and 5 μM concentrations. For BOLD-100, Ru uptake profiles overlapped substantially between WT and OxR cells at 100 μM, suggesting that oxaliplatin resistance has only a modest effect on BOLD-100 accumulation compared to oxaliplatin uptake. A detailed comparison is within Figure S4.

#### 3.2.4 Signal Spillover and Practical Considerations

In any barcoding single-cell experiment, potential signal spillover must be considered. This phenomenon challenges accurate debarcoding, as a fraction of segmented cells will exhibit low-level signals in a second barcode channel. Spillover can have multiple reasons beyond the application of barcoding protocols.^34–36^ Lastly, it can also concern the metal drug signals at the single-cell level, which require case-by-case evaluation. Oxaliplatin and BOLD-100 were selected for this proof-of-principle study, as these anticancer drugs bind to cellular macromolecules, minimizing drug loss during sample preparation.^20,21^ Moreover, spectral interferences were excluded by proper selection of barcode isotopes. ^196^Pt was selected to minimize the spectral interference arising from the high-abundant ^193^Ir-intercalator signal (see Figure S5–Figure S7). Generally, in LA-ICP-TOF-MS imaging at single-cell resolution, accurate signal assignment to segmented cells is inherently limited by the finite pixel resolution (e.g., 1 μm for the samples within this paper) and the corresponding lateral signal spillover, i.e., partial bleeding of signal from one cell into adjacent segmentation masks (Figure S8B). A second bleeding problem in IMC is line shift artifacts, as illustrated in Figure S8A. The highlighted red cell (^149^Sm) exhibits a ^151^Eu signal (green) due to a line shift causing bleed into the neighboring mask.

### 3.3 Phenotyping Compatibility of Barcodes

Finally, the barcoding strategy was evaluated for compatibility with phenotyping/IMC staining protocols to ensure that barcode integrity and sample traceability were preserved throughout the antibody staining. Otherwise, partial barcode loss, detachment, or redistribution could impair accurate sample assignment and compromise downstream analysis.

In Figure 4, 10 different experiments were barcoded using the dual-labeling approach (conditions in Table S1: 10-barcoded sample). Figure 4A shows cytospins without antibody staining, whereas Figure 4B presents a technical replicate that underwent a full immunostaining workflow to assess its impact on barcode stability.

**Figure 4:**
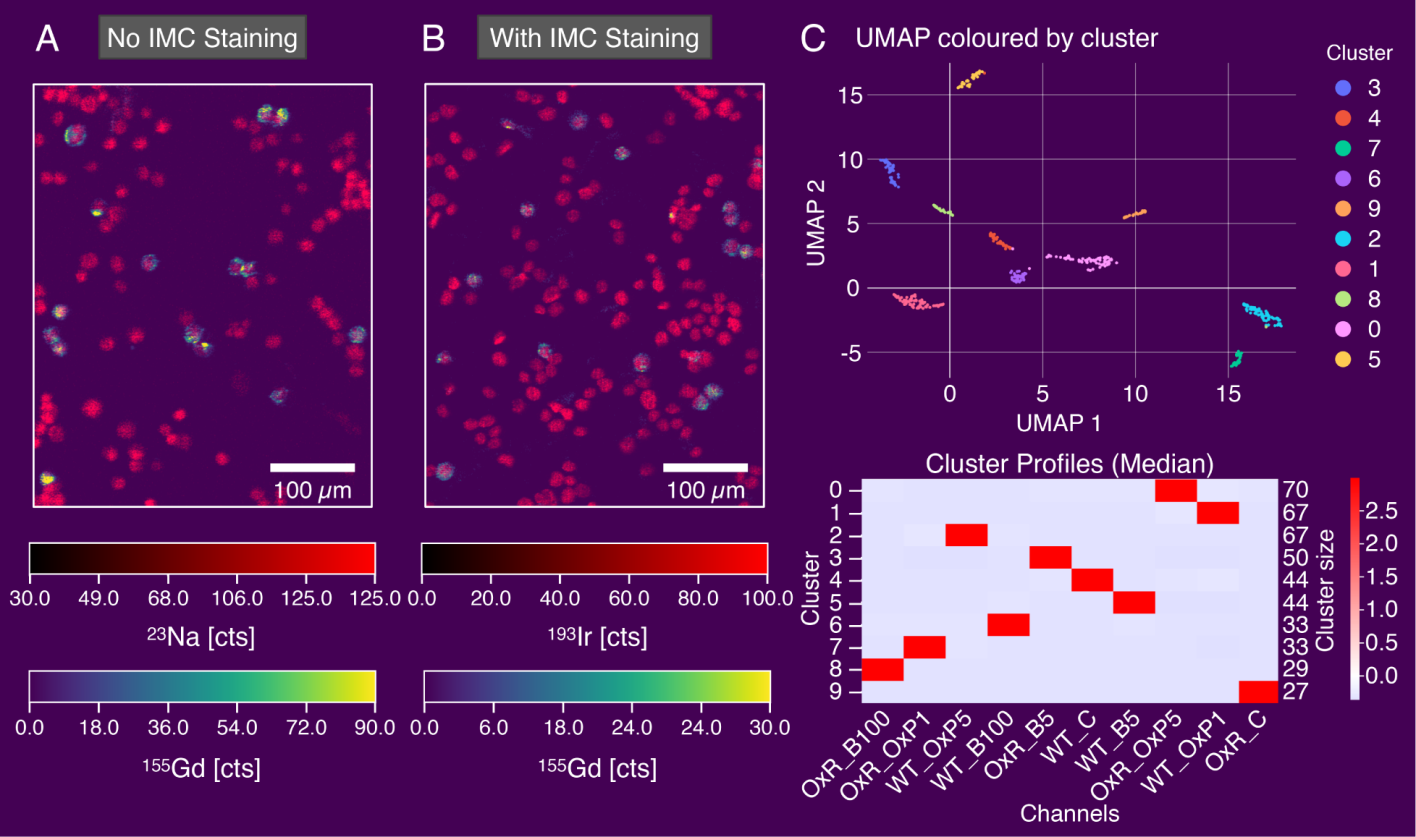
Cropped images of a nonantibody-stained sample (A) and an antibody-stained sample (B) illustrate the impact of antibody staining on barcode stability and the accurate retraceability of individual experimental conditions. (C) Debarcoding was performed by unsupervised Leiden clustering by phenograph and visualization via UMAP embedding, demonstrating the stability and retraceability of the barcode following antibody staining. Maps were acquired with LA-ICP-TOFMS at 1 μm resolution (2 μm circular spot size, 2× overlap in both directions) with a repetition rate of 500 Hz.

As illustrated in Figure 4, antibody staining was accompanied by a moderate decrease in the barcode signal intensity. This is primarily attributable to the heat-induced antigen retrieval step and subsequent washing steps. Antigen retrieval partially reverses fixation-induced cross-links and can alter protein conformations, which likely weakens the preformed WGA/anti-WGA complex and leads to partial barcode loss. Thus, a partial barcode loss was expected but neither impacted nor biased segmentation or debarcoding. In this respective data set, debarcoding is still ensured when using unsupervised Leiden clustering via PhenoGraph, even for small cell numbers (Figure 4C).

## 4 Conclusions

In this study, we introduce a robust single-isotope barcoding strategy for IMC and LA-ICP-TOFMS that enables multiple experimental conditions to be pooled into a single measurement while simultaneously improving the segmentation and data quality. By employing a dual-label WGA/anti-WGA complex as both a barcode carrier and a membrane marker, the workflow provides a high-intensity, spatially stable signal that supports reliable single-cell segmentation, facilitates the exclusion of doublets and missegmented objects, and remains traceable throughout fixation, antigen retrieval, and antibody staining.

The optimized barcoding conditions showed minimal to no spillover for both BOLD-100 and oxaliplatin. For other compounds, barcoding conditions may require additional optimization, as spillover susceptibility is highly dependent on the drug’s chemical properties. Unsupervised Leiden clustering proved to be an efficient and reproducible method for debarcoding, outperforming manual gating in scalability and in reducing user bias.

The applicability of the workflow was demonstrated in a multicondition study investigating oxaliplatin and BOLD-100 uptake in HCT116 cells, where barcoding enabled direct comparison across 10 conditions within a single measurement run. While the biological findings serve primarily as a proof of principle, the results illustrate the ability of the approach to generate consistent single-cell data sets across multiple experimental conditions.

Overall, the presented barcoding strategy decreases consumable usage, minimizes handling and batch effects, and increases analytical throughput while maintaining high segmentation and quantification quality. Its reliance on commercially accessible reagents and transparent chemistry makes it broadly applicable and readily extendable to other IMC workflows requiring high-resolution, multicondition single-cell analysis.

## Supplementary Material



## Data Availability

The data supporting the findings of this study are provided in the Supporting Information accompanying this article. Upon reasonable request, raw images of the data will be made available. For further inquiries, please contact Gunda Koellensperger at gunda.koellensperger@univie.ac.at.

## Notes

WGA/A-WGA concentration series, platinum and ruthenium washout compared in multiple setups, detailed view on BOLD-100 and oxaliplatin uptake, spectral interference for distinct Pt isotopes, experiments and treatments, instrumental parameters (PDF)
