# Supplementary material for: A Barcoding Strategy for Pooled Single-Cell LA-ICP-TOFMS Analysis of Metal-Containing Therapeutics
Source: Anal Chem. 2026 Jun 29;98(27):20352–60. doi: 10.1021/acs.analchem.6c01916 (PMC13373926; doi:10.1021/acs.analchem.6c01916)
Supplement: Supplementary file 1 [file ac6c01916_si_001.pdf]

Supporting Information

A barcoding strategy for pooled single-cell LA-ICP-TOFMS analysis of metal containing therapeutics

Claude Molitor[1-3], Lyndsey Hendriks[1], Antonia Hafner[4], Bernhard Keppler[2], Walter Berger[4], Gunda Koellensperger[1]*

[1] Institute of Analytical Chemistry, Faculty of Chemistry, University of Vienna, 1090 Vienna, Austria

[2] Institute of Inorganic Chemistry, Faculty of Chemistry, University of Vienna, 1090 Vienna, Austria

[3] Vienna Doctoral School in Chemistry (DoSChem), University of Vienna, 1090 Vienna, Austria

[4] Center for Cancer Research and Comprehensive Cancer Center, Medical University of Vienna, 1090 Vienna, Austria

* Corresponding authors:

Gunda Koellensperger

Institute of Analytical Chemistry, 1090 Vienna, Austria

Tel: +43-1-4277-52303, Email: gunda.koellensperger@univie.ac.at

Table of Contents

FIGURES S1 - S2 3
DETAILS TO THE BIOLOGICAL STUDY CASE 5
FIGURES S3 – S4 6
SPECTRAL INTERFERENCE FOR DISTINCT PT ISOTOPES 8
FIGURES S5 - S8 10
TABLE S1-S2 14

Figures S1 - S2

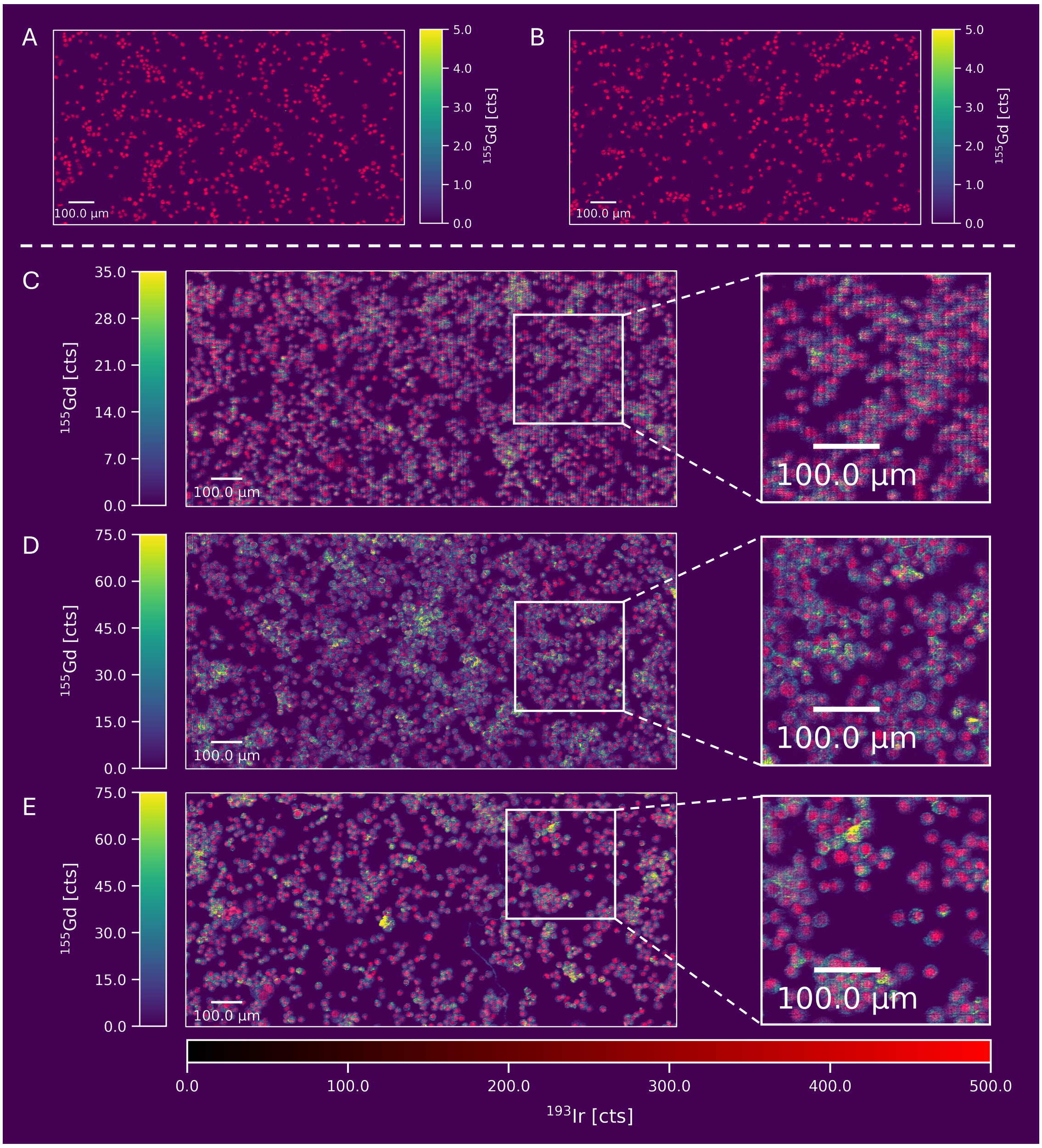

Figure S1: Optimization of the barcode concentration. One HCT116 WT cell culture was treated with 5 µM oxaliplatin for 16h and afterwards split into five aliquots. The non-barcoded sample A was washed and cytospins were made. Sample B represents a procedural control having undergone the barcoding procedure with buffers only. Samples C, D; and E were barcoded with 1, 3 and 5 µL ^{155}Gd-anti-WGA respectively (for 1.5 x 10^6 cells). The control (A) and procedural control (B) show no ^{155}Gd-anti-WGA signal. Best results were achieved with sample D (3 µL of ^{155}Gd-anti-WGA for 1.5 x 10^6 cells; equivalent for 15 cytopsins), providing a strong and uniform membrane signal. Maps were acquired with LA-ICP-TOFMS at 1µm resolution (3 µm Spots size, 3× overlap in both directions) with a repetition rate of 500 Hz.

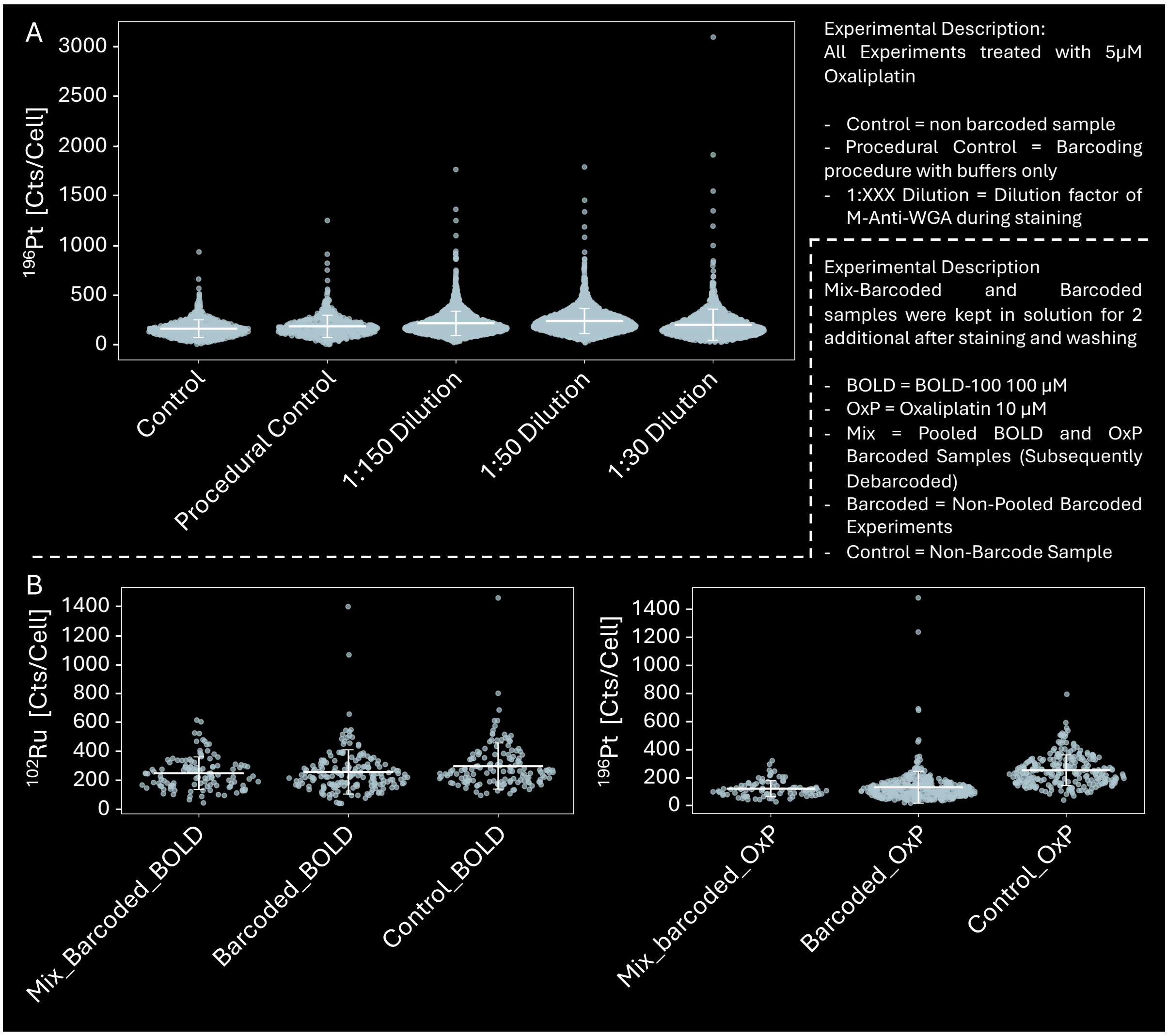

Figure S2: **A:** HCT116 WT cells treated with 5 µM Oxaliplatin. Comparison of ^{196}Pt-Washout within HCT116 WT cells in a non-barcoded sample (control), a procedural control undergoing the optimized barcoding procedure with buffers only and three different dilutions of M-Anti-WGA during barcoding. No difference between samples can be observed. **B:** HCT116 WT cells treated with 10 µM oxaliplatin (left) or 100 µM BOLD-100 (right). Comparison of the ^{102}Ru and ^{196}Pt washout in pooled barcoded and non-pooled barcoded samples, both remaining for 2 additional hours within cell suspension before making of the cytospins in contrast to a non-barcoded samples which was immediately cytospun after treatment. For BOLD-100, no significant washout is observed in contrast to Oxaliplatin.

Details to the biological study case

Ten experiments were conducted, individually barcoded, polled, cytospun, stained with an Ir-DNA-intercalator and measured using LA-ICP-TOFMS. Details are within Table S1 (10-barcoded sample). Segmentation was performed on the ^{193}Ir-DNA- and the stacked barcode signals. Cells touching the border were removed. Debarcoding was performed using unsupervised Leiden clustering by phonograph using MeXpose. Ambigious cells were removed.

For oxaliplatin, clear differences were observed between the two cell lines. At 1 µM oxaliplatin, WT cells showed a median ^{196}Pt signal of 19.0 cts/cell, whereas HCT116 OxR cells displayed only 8.7 cts/cell (-54,2 %). This difference became more pronounced at 5 µM, where WT cells reached a median ^{196}Pt-content of 71.8 cts/cell compared to 26.3 cts/cell in OxR cells (-63,4 %). Control and BOLD-100 treated samples showed comparable ^{196}Pt background levels across both lines (≈4-5 cts/cell) due to spectral interference (Ir-staining) which could be used for background correction. Together, these results confirm that the OxR phenotype is associated with a substantial reduction in intracellular platinum accumulation.

In contrast, uptake of the ruthenium-based drug BOLD-100 differed less between WT and OxR cells. At 5 µM BOLD-100, median ^{102}Ru intensities were nearly identical (WT median: 13.3 cts/cell; OxR median: 12.3 cts/cell). At 100 µM, both cell lines showed elevated ^{102}Ru accumulation, with median values of 416.5 cts/cell in WT and 308.0 cts/cell in OxR cells, corresponding to a 26,1 % decrease in the OxR. Although some differences in the upper intensity range were observed at 100 µM, the overall Ru uptake profiles overlapped substantially between WT and OxR cells, suggesting that oxaliplatin resistance has only a modest effect on BOLD-100 accumulation compared to the Oxaplatin uptake.

Using this study case to illustrate the applicability of our barcoding strategy, we were able to demonstrate a key distinction between the two drugs: platinum uptake is substantially impaired in OxR cells, whereas BOLD-100 accumulation shows only minor differences between WT and OxR, indicating that BOLD-100 is less affected by the reduced-drug-accumulation phenotype characteristic of oxaliplatin resistance.

Figures S3 – S4

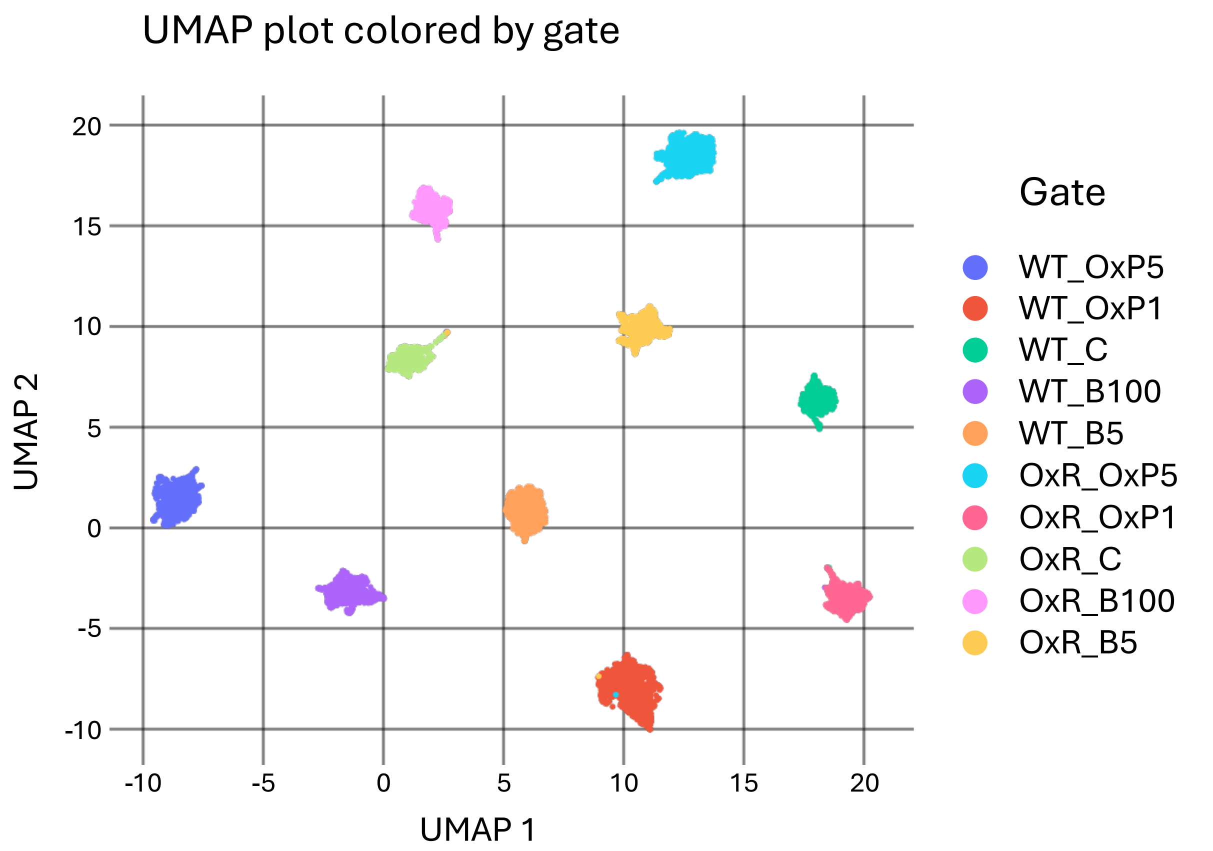

Figure S3: Ten individually barcoded and subsequently pooled experiments of HCT116 wild-type (WT) and oxaliplatin-resistant (OxR) cells treated with oxaliplatin (OxP) and BOLD-100 (B) at the indicated concentrations (concentration in µM as number behind the metallodrugs) for 16h. Debarcoding was performed by density-plot-based manual gating. Ambiguous cells were removed during post-processing. UMAP was computed based on the 10 barcode channels and colored by manual gate assignment. Clear and consistent separation of experiments can be observed.

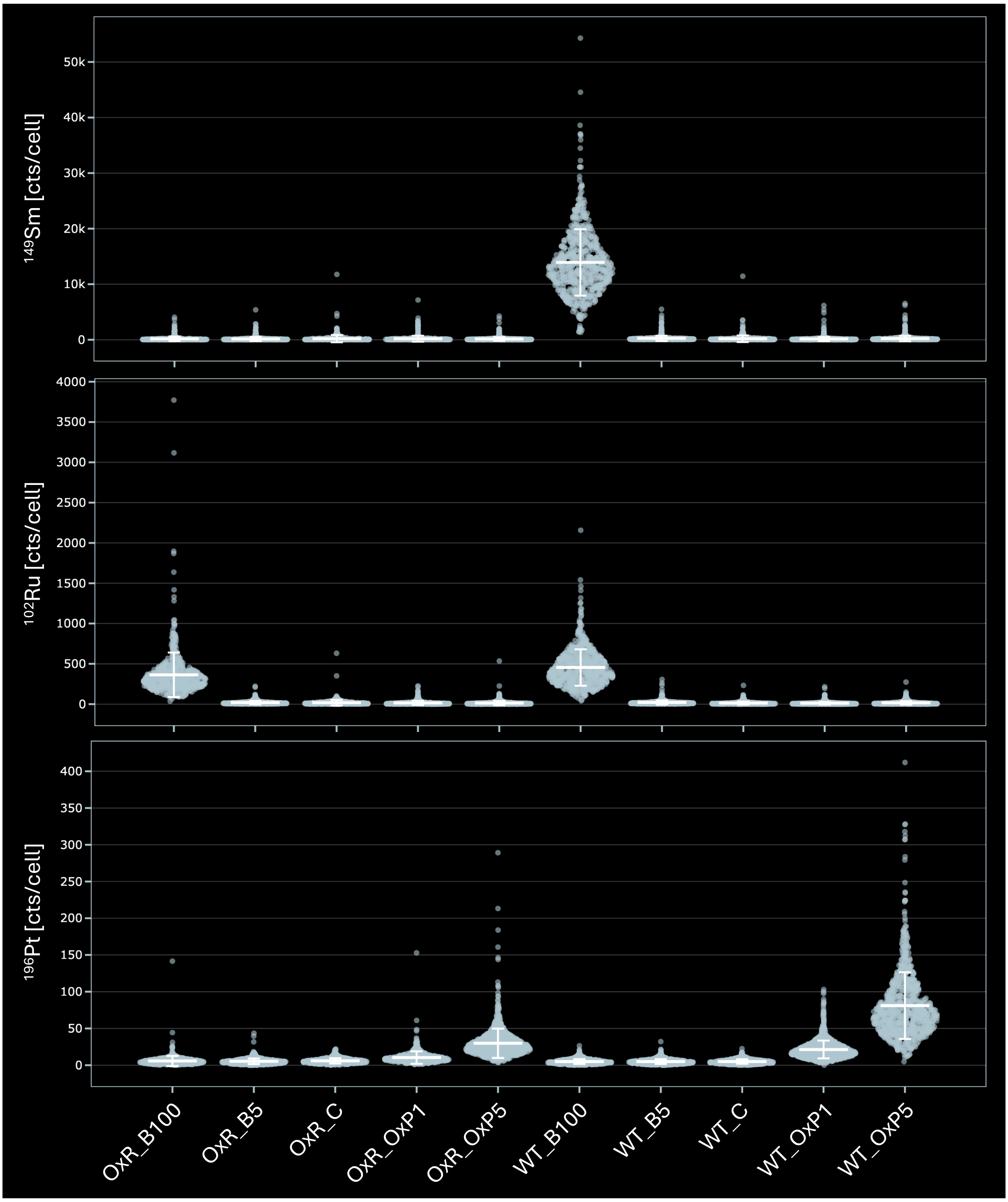

Figure S4: Ten individually barcoded and subsequently pooled experiments of HCT116 wild-type (WT) and oxaliplatin-resistant (OxR) cells treated with oxaliplatin (OxP) and BOLD-100 (B) at the indicated concentrations (concentration in µM as number behind the metallodrugs) for 16h. Samples were segmented with the stacked barcode signal and the 193Ir (DNA-intercalator). Debarcoding was performed using usupervised Leiden Clustering. Ambiguous cells were removed. Violoin plots of ^{149}Sm as exemplary barcode, ^{102}Ru and ^{196}Pt.

Spectral interference for distinct Pt isotopes

When Ir-intercalator staining is performed, a substantial signal can emerge at m/z = 194 that scales with the ^{193}Ir DNA-intercalator intensity. Spectral interferences in imaging mass cytometry may arise from background elevation due to intense neighboring peaks, from polyatomic or adduct formation in the collision/reaction cell, or due to contamination of reagents or consumables. Such artifacts are well known in mass cytometry and are not specific to barcoded samples.

To illustrate this phenomenon and support the choice of using the ^{196}Pt for oxaliplatin comparisons within this paper, HCT116 cells treated with 100 µM BOLD-100 for 16h were analyzed. These samples contained no platinum-based compound; therefore, any Pt-channel signal must result from interference. Figure S5 compares two technical replicates with different Ir intensity. Apparent ^{194}Pt and ^{196}Pt signals increased with increasing ^{193}Ir signal, indicating an Ir-related origin rather than true platinum contamination.

To further verify the origin of the apparent ^{194}Pt signal, the same cytospin of BOLD-100-treated cells was analyzed in both CCT and STD mode (Figure S6). Within the ^{193}Ir channel, the DNA-intercalator signal showed an exceptionally high intensity due to extended staining, providing a clear visualization of cell morphology. Comparison of the 194 m/z channel revealed that the apparent ^{194}Pt signal observed in CCT mode was dramatically reduced when measured in STD mode, consistent with the formation of an ^{1}H-^{193}Ir adduct in the CCT mode. Background subtraction further diminished this apparent signal, confirming that part of the elevation originates from baseline background rather than true platinum. A direct comparison of both acquisition modes, with and without background correction, demonstrates that the observed 194 m/z intensity results from gas-phase adduct formation rather than from a genuine Pt signal. Peak fitting and baseline subtraction was applied in TOFWARE 3.2.3 to reduce peak tailing on adjacent masses.[1] The remaining signal after background subtraction might possibly be a Pt impurities within the DNA intercalator which is also mentioned by Standard Biotools in their sample description.

Additionally, a dilution series of the dual-label (see Figure S7), was used to further investigate the influence of the Ir-DNA intercalator on the apparent platinum isotopes signal. Colon cancer cells (HCT116) were exposed to 5 µM oxaliplatin for 16 hours. Two aliquots were used for quality control as procedural blanks: a non-barcoded sample and a sample prepared following the barcoding steps but using buffers only (Figure S1 A and B). The remaining three aliquots comprised a three-point concentration series, with final M-anti-WGA dilutions of 1:150; 1:50; and 1:30 during the 30-minute barcoding incubation (Figure S1 C; D and E). When comparing platinum isotopes within these samples (Figure S7), the ratios ^{194}Pt /^{195}Pt, ^{194}Pt/^{196}Pt, and ^{192}Pt/^{196}Pt deviated from their respective natural abundances (Figure S7 B). Only the ^{195}Pt/^{196}Pt ratio matched the expected natural ratio for all samples, confirming that interferences predominantly affect masses adjacent to ^{193}Ir. For this reason, the ^{196}Pt isotope was selected for all subsequent analyses, as it is least likely to be compromised by Ir-related spectral artifacts. At laser repetition rates exceeding 300 Hz, the acquisition speed of the ICP-TOFMS used in this study becomes limiting, such that full mass spectra cannot be recorded. Instead, only temporally integrated ion signals (“stick data”) are saved to keep pace with the ablation rate. Because the baseline signal is not recorded under these conditions, background characterization

and subtraction are not possible. In contrast, at repetition rates of 300 Hz or below, full-profile mass spectra can be acquired with sufficient temporal resolution to capture baseline ions, enabling reliable background subtraction.[1] Finally, the Ir-DNA-intercalator, diluted 1:100 within TBS was analyzed. An apparent signal at m/z 192 and 194, 195, and 196 appears that is not present in STDS mode. (Figure S7 C)

While spectral interferences can be recognized and mitigated by adjusting the acquisition speed for baseline subtraction or monitoring alternative platinum isotopes using the multi-element detection capability of TOFMS, future studies may benefit from replacing Ir-based DNA intercalators with Rh-based alternatives to avoid this interference altogether.

(1) Hendriks, L.; Gundlach-Graham, A.; Hattendorf, B.; Günther, D. Characterization of a New ICP-TOFMS Instrument with Continuous and Discrete Introduction of Solutions. *J. Anal. At. Spectrom.* **2017**, *32* (3), 548–561. https://doi.org/10.1039/C6JA00400H.

Figures S5 - S8

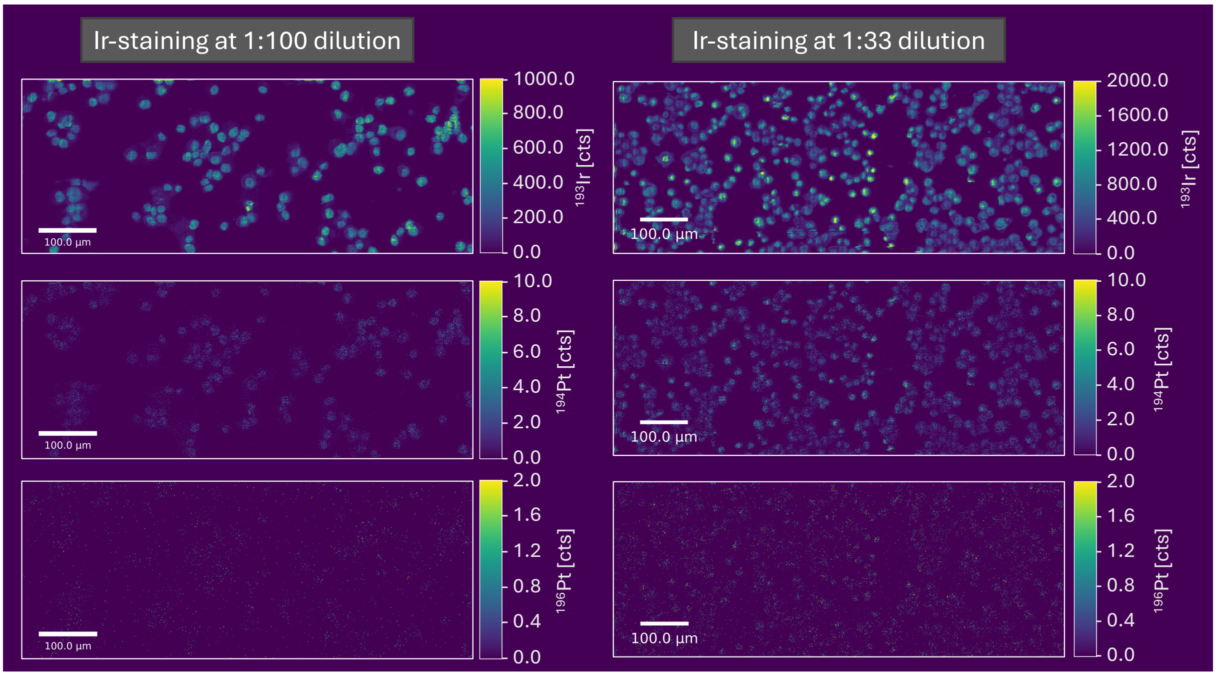

Figure S5: Two technical replicates of HCT116 WT cell treated with BOLD-100 at 100 µM. No Platinum based drug was used. Comparison of an apparent signal at m/z 194 between two samples stained at different Ir-DNA-Intercalator dilutions. The apparent signal at m/z 194 is more intense for the sample with the elevated ^{193}Ir signal. Maps were acquired with LA-ICP-TOFMS at 1µm resolution (2 µm circular spot size, 2× overlap in both directions) with a repetition rate of 500 Hz.

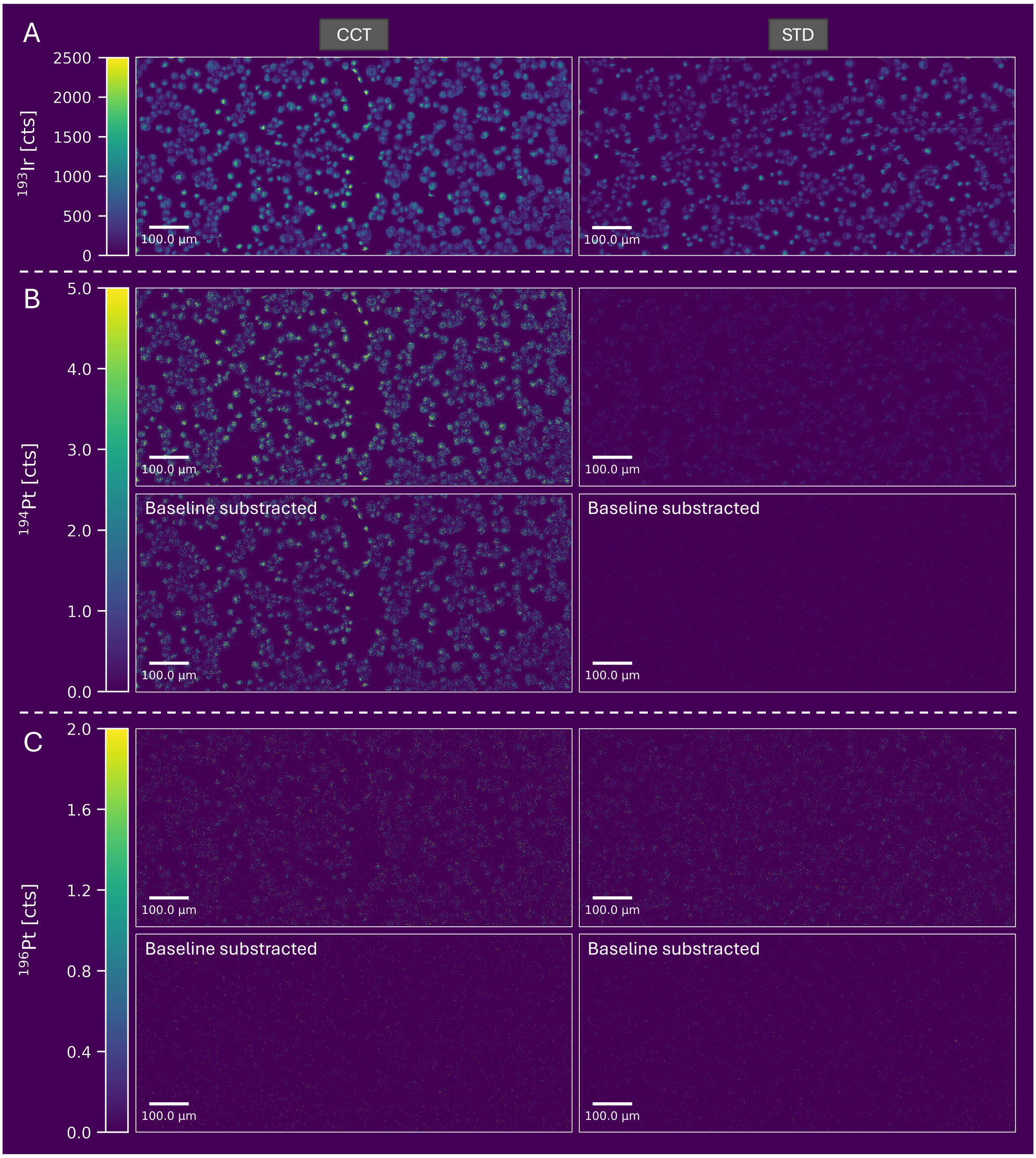

Figure S6: Comparison of the same cytospin (HCT116 WT cells treated with BOLD-100 at 100 µM) analyzed in CCT and STD mode. (A) ^{193}Ir-DNA intercalator visualizing cell morphology. The image shows an exceptionally high Ir intensity due to extended Ir-DNA-intercalator staining. (B) Comparison of the 194 m/z channel acquired in CCT and STD mode, including the background-subtracted version. The apparent ^{194}Pt signal observed in CCT mode is dramatically reduced in STD mode, consistent with the formation of an ^{1}H-^{193}Ir adduct in the CCT mode. Background correction further decreases this apparent signal, confirming a background-induced component. (C) Direct comparison of the 196 m/z channel across both acquisition modes with and without background subtraction, illustrating that the observed intensity originates from gas-phase adduct formation rather than true platinum signal. Maps were acquired with LA-ICP-TOFMS at 1µm resolution (2 µm circular spot size, 2× overlap in both directions) with a repetition rate of 300 Hz.

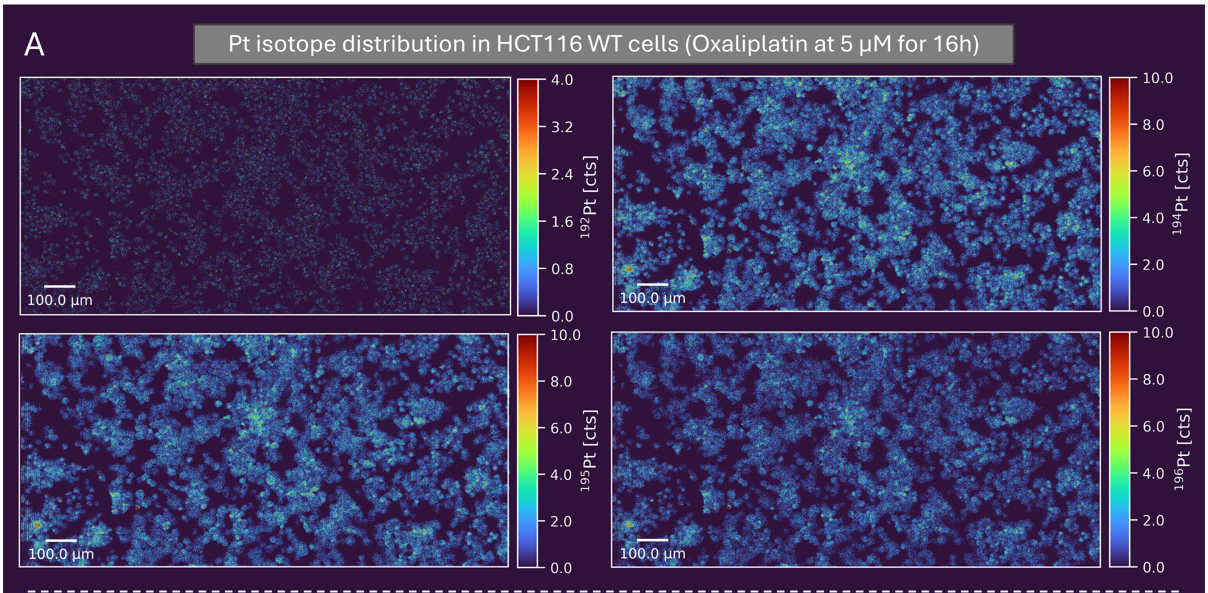

B

	Mean				Median	
Isotope ratio:	194/196	194/195	195/196	192/196	194/196	195/196
Ctr	1,729	1,291	1,339	0,343	1,707	1,340
Procedural control	1,690	1,272	1,329	0,320	1,681	1,334
1:150 M-Anti-WGA	1,539	1,157	1,330	0,214	1,540	1,333
1:50 M-Anti-WGA	1,449	1,087	1,333	0,152	1,446	1,333
1:30 M-Anti-WGA	1,604	1,198	1,338	0,254	1,644	1,355
Theoretical ratio:	1,300	0,973	1,336	0,031	1,300	1,336

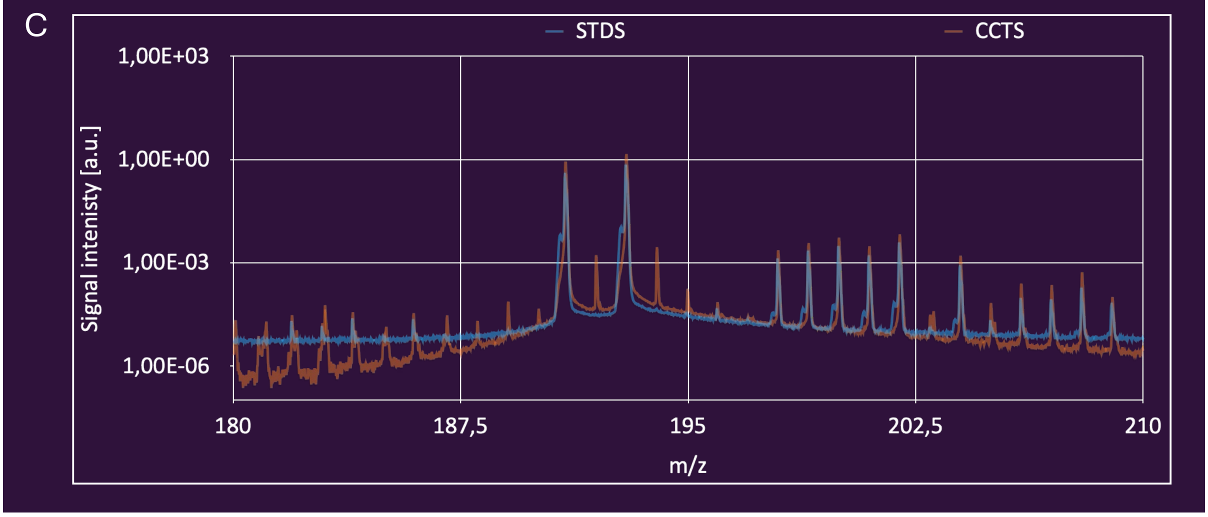

Figure S7: **A:** HCT116 WT cells treated with oxaliplatin at 5 µM for 16h and barcoded with ^{155}Gd-Anti-WGA at a 1:50 dilution. Maps of four Pt isotopes for isotope ratio comparisons. Maps were acquired with LA-ICP-TOFMS at 1µm resolution (2 µm circular spot size, 2× overlap in both directions) with a repetition rate of 500 Hz. **B:** HCT116 WT cells treated with 5 µM oxaliplatin for 16h. Samples represent a non-barcoded sample (ctr), a procedural control undergoing the optimized barcoding procedure with buffers only and three different dilutions of ^{155}Gd-Anti-WGA during barcoding. Cells were segmented with the ^{155}Gd and ^{193}Ir (DNA) signal. Theoretical isotope ratios compared to the isotope ratios within the sample illustrating apparent signals at ^{192}Pt and ^{194}Pt lead to wrong isotope ratios. ^{195}Pt/^{196}Pt is the only isotope ratio which matches the theoretical one in all five samples. **C:** Measurement of the Ir-Intercalator, diluted 1:100 within TBS. An apparent signal at m/z 192 and 194, 195, and 196 appears which are not present in STDS mode.

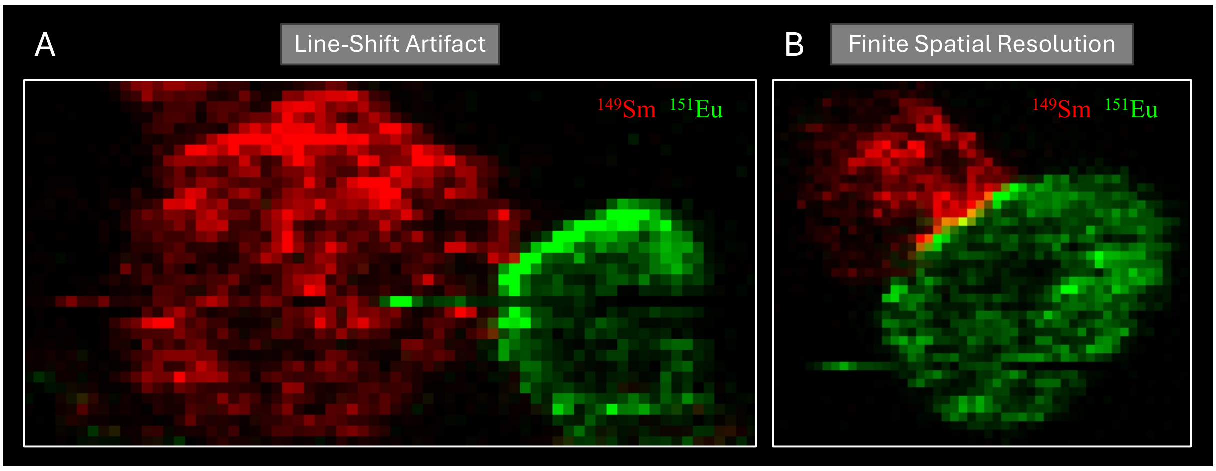

Figure S8: HCT116 cells barcoded with once ^{151}Eu-anti-WGA ^{149}Sm-anti-WGA (A) Example of a line-shift artifact. The line artifact leads to a ^{151}Eu-signal within the red ^{149}Sm-barcoded cell. During segmentation and data extraction the red cell shows an ^{151}Eu-signal. (B) Example illustrating finite spatial resolution. Pixels at cell-cell boundaries must be assigned to one leading to a signal bleed into the neighboring cell. Maps were acquired with LA-ICP-TOFMS at 1µm resolution (2 µm circular spot size, 2× overlap in both directions) with a repetition rate of 500 Hz.

Table S1-S2

Table S1: Experiments and treatments.

Experiments	HCT116	Treatment	Abr.	Treatment Time [h]	Metal label	Anti-WGA volume [µL]*	Pooled
**10-barcode sample**	WT	Control	WT_C	16	^{143}Nd	5	Pooled
		Oxaliplatin 1 µM	WT_OxP1	16	^{144}Nd	5	
		Oxaliplatin 5 µM	WT_OxP5	16	^{154}Sm	5	
		BOLD-100 5 µM	WT_B5	16	^{147}Sm	5	
		BOLD-100 100 µM	WT_B100	16	^{149}Sm	5	
	OxR	Control	OxR_C	16	^{151}Eu	5	
		Oxaliplatin 1 µM	OxR_OxP1	16	^{152}Sm	5	
		Oxaliplatin 5 µM	OxR_OxP5	16	^{145}Nd	5	
		BOLD-100 5 µM	OxR_B5	16	^{155}Gd	5	
		BOLD-100 100 µM	OxR_B100	16	^{165}Ho	5	
**Washout**	WT	BOLD-100 100 µM	/	16	-	-	
		Oxaliplatin 10 µM	/	16	-	-	
		BOLD-100 100 µM	/	16	^{143}Nd	7.5	Pooled and individual
		Oxaliplatin 10 µM	/	16	^{155}Gd	7.5	
**Concentration series**	WT	Oxaliplatin 5 µM		16	^{155}Gd	5	
		Oxaliplatin 5 µM		16	^{155}Gd	3	
		Oxaliplatin 5 µM		16	^{155}Gd	1	
		Oxaliplatin 5 µM		16	-	0**	
		Oxaliplatin 5 µM		16	-	-	
**Experiments**	**HELA**	**Condition**			**M-Anti-WGA**	**Anti-WGA volume [µL]***	**Pooled**
**Specificity sample**	WT	Ctr Living	/	0	^{149}Sm	7.5	Pooled
		Ctr Fixed	/	0	^{151}Eu	7.5	
		DTX Treated Living	/	72	^{152}Sm	7.5	
		DTX Treated Fixed	/	72	^{165}Ho	7.5	

*Volume per 1,5 x 10^6 cells used **procedural control using buffers only during the barcoding procedure

Table S2: LA-ICP-TOFMS parameters

Parameter	
ICP-TOFMS	
RF Power [W]	1440
Sampling depth [mm]	2.9
Cone materials	Ni
Plasma gas flow [L min^{-1}]	14
Auxiliary gas flow [L min^{-1}]	0.80
Nebulizer gas flow [L min^{-1}]	1.06
Measurement mode	Collision cell technology (CCT)<br>Standard mode (STD)
CCT gas	93% He (*v*/*v*), 7% H2 (*v*/*v*)
CCT gas flow [mL min^{-1}]	4.2
m/z range	14-256
**Laser ablation**	
Spot size	2-3 µm (circular)
Interspacing (Y - Overlap)	1 µm
Repetition rate	300-500 Hz
Dosage (X - Overlap)	2-3
Shot count	1
Fluence	0.6 - 1.2 J cm^{-2}
